# Untangling the Uncertain Role of Overactivation of the Renin–Angiotensin–Aldosterone System with the Aging Process Based on Sodium Wasting Human Models

**DOI:** 10.3390/ijms25179332

**Published:** 2024-08-28

**Authors:** Chantelle Thimm, James Adjaye

**Affiliations:** 1 Institute for Stem Cell Research and Regenerative Medicine, Medical Faculty, Heinrich-Heine University Düsseldorf, 40225 Düsseldorf, Germany; 2 Zayed Centre for Research into Rare Diseases in Children (ZCR), EGA Institute for Women’s Health, University College London (UCL), 20 Guilford Street, London WC1N 1DZ, UK

**Keywords:** sodium deficiency diseases, distal convoluted tubules, renin–angiotensin–aldosterone system, ageing, sodium accumulation

## Abstract

Every individual at some point encounters the progressive biological process of aging, which is considered one of the major risk factors for common diseases. The main drivers of aging are oxidative stress, senescence, and reactive oxygen species (ROS). The renin–angiotensin–aldosterone system (RAAS) includes several systematic processes for the regulation of blood pressure, which is caused by an imbalance of electrolytes. During activation of the RAAS, binding of angiotensin II (ANG II) to angiotensin II type 1 receptor (AGTR1) activates intracellular nicotinamide adenine dinucleotide phosphate (NADPH) oxidase to generate superoxide anions and promote uncoupling of endothelial nitric oxide (NO) synthase, which in turn decreases NO availability and increases ROS production. Promoting oxidative stress and DNA damage mediated by ANG II is tightly regulated. Individuals with sodium deficiency-associated diseases such as Gitelman syndrome (GS) and Bartter syndrome (BS) show downregulation of inflammation-related processes and have reduced oxidative stress and ROS. Additionally, the histone deacetylase sirtuin-1 (SIRT1) has a significant impact on the aging process, with reduced activity with age. However, GS/BS patients generally sustain higher levels of sirtuin-1 (SIRT1) activity than age-matched healthy individuals. SIRT1 expression in GS/BS patients tends to be higher than in healthy age-matched individuals; therefore, it can be assumed that there will be a trend towards healthy aging in these patients. In this review, we highlight the importance of the hallmarks of aging, inflammation, and the RAAS system in GS/BS patients and how this might impact healthy aging. We further propose future research directions for studying the etiology of GS/BS at the molecular level using patient-derived renal stem cells and induced pluripotent stem cells.

## 1. Introduction

The kidney is a paired organ associated with the urinary system and performs numerous tasks such as filtration of urine and regulation of homeostasis of electrolytes such as sodium, potassium, calcium, magnesium, and phosphate [1].

The processes involved in the regulation of sodium and water balance, including blood pressure and the glomerular filtration rate, have a decisive influence on sodium and water transport in the renal tubule. An imbalance is accompanied by undesirable consequences. Several diseases are associated with sodium deficiency; these include Gitelman syndrome (GS) and Bartter syndrome (BS) [2,3,4,5,6].

Gitelman syndrome is an autosomal recessive tubular disorder caused by biallelic mutations in a gene encoding the protein that is a sodium–chloride cotransporter. These are responsible for approximately 7–10% of the tubular absorption of electrolytes. GS is characterized by an increased excretion of magnesium (hypermagnesiuria) and a reduced excretion of calcium (hypocalciuria) in the urine. In addition, GS patients have hypokalemic alkalosis, i.e., a disorder of the acid–base balance and salt loss. Other signs of illness include muscle weakness, fatigue, cramps, and low blood pressure caused by an electrolyte imbalance due to distinct mutations within the SLC12A3 encoding gene [7,8,9].

Each individual, at some point, encounters the progressive biological process of aging associated with oxidative stress, senescence, and reactive oxygen species (ROS). GS/BS patients show downregulation of inflammation-related processes and have reduced oxidative stress and ROS. In this context, GS and BS patients, in comparison to healthy individuals, sustain higher levels of sirtuin-1 (SIRT1) activity, which seems to have a significant impact on hypertension and the aging process [10,11,12].

We highlight the importance of the hallmarks of aging and the renin–angiotensin–aldosterone system (RAAS) in Gitelman syndrome patients and how this might impact healthy aging.

## 2. Gitelman Syndrome

The nephrologist Hillel Jonathan Gitelman first observed patients with various conditions associated with metabolic alkalosis, electrolyte imbalance, and salt wasting in the late 1960s [8]. Gitelman syndrome (GS) (OMIM 263800) is one of the most common renal tubulopathies and is known as familial hypokalemia–hypomagnesemia. It is an autosomal recessive inherited disorder caused by a disturbance in the function of the thiazide-sensitive sodium chloride cotransporter (NCC) in the kidney. In this process, blood levels show magnesium and potassium deficiency, while the urine has high levels of magnesium excretion and decreased excretion of calcium. GS is characterized by metabolic alkalosis, hypomagnesemia, and hypercalciuria. The etiology is a transport defect of the distal convoluted tubule (DCT). It is manifested in adolescence or adulthood and has an estimated prevalence of 1:40,000 Caucasian individuals [13]. It is more prevalent in the Asian population and is estimated at around 1.7 per 1000 people [13]. The actual frequency of GS is difficult to determine in the general population due to its inconspicuousness or misdiagnosis.

The disease is caused by biallelic inactivating mutations within the SLC12A3 encoding gene, which encodes the NCC. Its genomic locus is chromosome 16q13 and it consists of 26 exons that code for 1002 amino acids [9,14,15].

NCC is expressed by cells of the apical membrane lining the distal convoluted tubule [9]. Studies have identified more than 350 distinct mutations within the SLC12A3 encoding gene in GS patients [16,17,18]. In addition to this, mutations in the cation channel subfamily 6 of the protein Claudin 16 gene (TRPM6), which is involved in magnesium transport in distal tubules [19,20].

**Figure 1:**
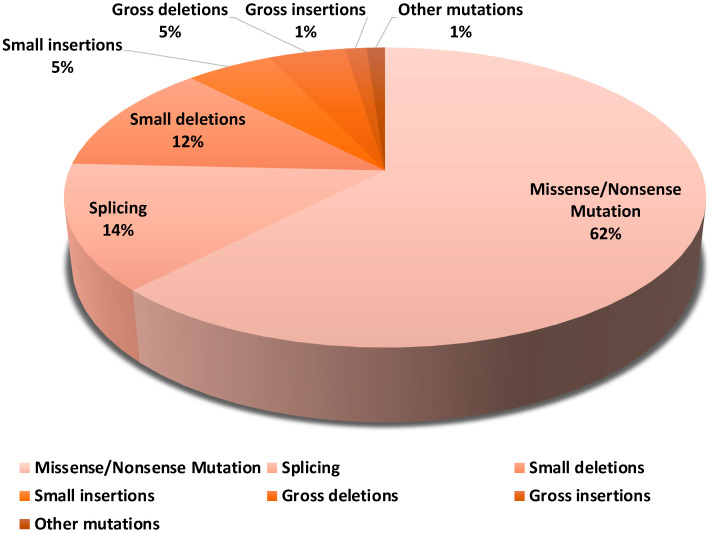
SLC12A3 mutation variants. Gitelman syndrome is caused by biallelic inactivating mutations in the SLC12A3 gene. It consists of 26 exons that code for 1002 amino acids. There are more than 350 different mutations that are caused by missense and nonsense variants, frameshift, splice, and deep intronic variants, as well as large genomic rearrangements. The pie chart represents all variants of mutations in the SLC12A3 gene. The largest population of mutations are missense and nonsense mutations, with a prevalence of approximately 62.1%. The splice mutations are present at approximately 14% and small deletions at around 12%. Major deletions and small insertions make up approximately 5%, whereas the remaining mutation types account for 7.4% [21].

The largest proportion of mutations in SLC12A3 is 62.1% missense and nonsense mutations. A missense mutation is when the error in spelling a single base pair creates a different amino acid. If the mutation is meaningless (nonsense mutation), a so-called stop codon results, and the synthesis of the protein is terminated. Furthermore, the splice mutations are present at around 13.5% and small deletions at around 12%. Finally, there are the major deletions and small insertions at 5%. Other mutations account for 7.4% (Figure 1) [18].

The most common mutation is heterozygous, which has a prevalence of 1% in Europe [21]. Studies have shown that homozygous mutations have also been identified in a Spanish cohort [22].

An example of multiple mutations was found in patients in Taiwan. Here, triple mutations in GS patients are presented with a prevalence of approximately 12%, which is certainly due to the higher frequency of heterozygous carriers within the Asian population [23].

The mutations lead to sodium efflux and activation of adaptive mechanisms such as the RAAS [24]. The NCC is the salt-reabsorptive transporter localized at the apical membrane in the DCT. It has been documented that RAAS is a primary physiological regulator of NCC [25]. The inactivation of NCC leads to the activation of RAAS, which results in reduced tubular reabsorption of sodium and chloride. This deficiency leads to dehydration, which triggers RAAS as a compensatory mechanism. Additionally, low levels of potassium in blood serum have been described, as well as an increase in the levels of renin and aldosterone.

Studies based on GS rodent models revealed that Ca^2+^ deficiency has significant consequences on DCT function as compromised NCC function leads to structural remodeling of DCTs [26]. The disease can be asymptomatic or associated with mild symptoms, such as weakness, fatigue, craving for salt, thirst, or nocturia. GS patients often have transient episodes of muscle weakness and tetany, sometimes combined with abdominal pain, vomiting, and fever [27,28]. The presence of hypocalciuria and hypomagnesemia has high predictive value for the clinical diagnosis of GS [29,30].

If GS is suspected, there are not only clinical complaints but also biochemical criteria, which include chronic hypokalemia (<3.5 mmol/L) and renal potassium loss, determined as a spot urine sample with a potassium/creatinine ratio >2.0 mmol/mol (>18 mmol/g) [31].

Hypocalciuria is determined as a spontaneous urine calcium/creatinine ratio <0.2 mmol/mmol (0.07 mg/mg). GS patients have low urinary calcium excretion [25,32], high fractional magnesium excretion, and hypomagnesemia [8,25].

Metabolic alkalosis, hypomagnesemia <0.7 mmol/L (<1.70 mg/dL) [33], and renal magnesium loss (fractional magnesium excretion >4%) occur in Gitelman syndrome [34]. Gitelman syndrome occurs in both males and females alike.

In adults, additional criteria include fractional chloride excretion >0.5%, normal or low blood pressure, and the absence of morphologic and functional renal abnormalities [11,31] including increased renin activity and aldosterone expression [35].

GS is caused by a variety of mutations within the genes encoding SLC12A3 (thiazide-sensitive sodium chloride cotransporter (NCC): HNF1B (hepatocyte nuclear factor 1β), FXYD2 (sodium/potassium-transporting ATPase gamma chain—a member of the FXYD family of transmembrane proteins), KCNJ10 (ATP-sensitive inward rectifier potassium channel 10, a member of the inward rectifier-type potassium channel family) [16,36,37,38,39]. EAST or SeSAME syndrome closely resembles Gitelman syndrome. This is an autosomal recessive disorder caused by mutations within the KCNJ10 gene. EAST is an acronym for epilepsy, ataxia, sensorineural deafness, and (a renal salt-wasting) tubulopathy, and SeSAME stands for seizures, sensorineural deafness, ataxia, mental retardation, and electrolyte imbalance [37,38,40].

Mitochondria are essential components of eukaryotic cells. Mitochondrial DNA (mtDNA) mutations can occur during mitosis, which then results in changes in the degree of heteroplasmy. If the proportion of mtDNA exceeds the threshold value of 60–80% [41], this results in a considerable drop in energy production, leading to the development of metabolic disorders. Pathogenic mtDNA variants of genes encoding the transfer RNAs for phenylalanine (MT-TF) and isoleucine (MT-TI) cause a Gitelman-like syndrome [42].

There is currently no specific therapy, but potassium and magnesium supplementation and a high-salt diet are recommended for all GS patients. To counteract disturbed hormone levels and fluid and electrolyte balance, spironolactone or triamterene are prescribed to GS patients to reduce potassium and magnesium losses [43]. In GS patients, a high dose of potassium chloride is used to combat hypokalemia. Salts with poor absorbable anions such as aspartan should not be administered to patients, as these do not lead to any improvement and may even exacerbate alkalosis.

Children, or even infants, are prescribed a nonsteroidal anti-inflammatory drug (NSAID) known as indomethacin, which is used to treat growth deficits in severe, early-onset forms of the disease [44,45].

## 3. Bartter Syndrome

In 1962, Bartter et al. identified a new syndrome and named it Bartter syndrome (BS) [46]. BS is one of the rare hereditary renal tubular disorders caused by impaired salt reabsorption in the thick ascending limb (TAL) of the loop of Henle. It is associated with several electrolyte abnormalities including low potassium and chloride, salt wasting, hypokalemia, and metabolic alkalosis with hyperaldosteronism with normal blood pressure and hyperplasia of the juxtaglomerular apparatus (JGA) [46,47].

**Figure 2:**
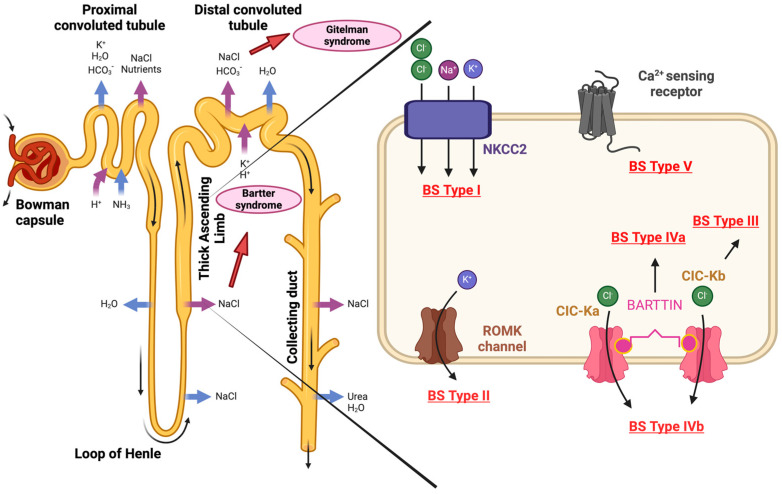
Mutations associated with Bartter syndrome. This is caused by homozygous or mixed heterozygous mutations in one of the following genes: SLC12A1, KCNJ1, CLCNKB, BSND, or CASR. Depending on the mutated gene, BS can be divided into five subtypes: BS type I—SLC12A1 codes for the sodium–potassium–chloride cotransporter NKCC2; BS type II—KCNJ1 codes for the potassium channel ROMK; BS type III—CLCNKB codes for chloride channel ClC-Kb; BS type IVa—BSND codes for barttin, which is a subunit of the chloride channels ClC-Ka and ClC-Kb; BS type IVb—simultaneous mutations in CIC-Ka and CIC-Kb; BS type V—CASR, which encodes the basolateral calcium receptor CASR. The Figure was created with BioRender.com.

In contrast to GS, Bartter syndrome is a more severe congenital kidney injury that often manifests itself earlier, usually within the first year of life [48]. BS is a polygenic disease caused by homozygous or mixed heterozygous mutations in one of the following genes: SLC12A1, KCNJ1, CLCNKB, BSND, or CASR (Figure 2).

Depending on the mutated gene and, thus, the affected transporter protein, BS can be divided into at least five subtypes, which are expressed in the tubular epithelial cells of the TAL of the Henle loop. Type I BS is characterized by loss-of-function mutations within the SLC12A1 gene and is known as neonatal Bartter syndrome (hyperprostaglandin E syndrome). This codes for the apical sodium–potassium–chloride cotransporter NKCC2. Type II of BS, also known as neonatal Bartter syndrome, is caused by loss-of-function mutations in the KCNJ1 gene, which codes for the apical inwardly rectifying potassium channel ROMK. BS type III, also known as classic Bartter syndrome, is caused by mutations in the CLCNKB gene, which codes for the basolateral chloride channel ClC-Kb. Distinct variants of chloride channels are present in most organs but CLCNKB, affected here, is found exclusively in the kidney, so the clinical manifestation of a mutation in this channel is only seen in the kidney [49].

Classical Bartter syndrome with sensorineural hearing loss (hyperprostaglandin E syndrome), or BS type IVa, is caused by loss-of-function mutations in the BSND gene, which encodes barttin, a β-subunit for ClC-Ka and ClC-Kb. Parallel to BS type IVa, simultaneous mutations in CIC-Ka and CIC-Kb are present in type IVb. Bartter syndrome type V with hypocalcemia is also known as autosomal dominant hypoparathyroidism. This is characterized by gain-of-function mutations in the Ca^2+^-sensing receptor CASR, which codes for the basolateral calcium receptor CAS.

Bartter syndrome is an inherited disorder of the renal tubules caused by impaired salt reabsorption. It leads to several electrolyte abnormalities including low potassium and chloride, salt wasting, hypokalemia, and metabolic alkalosis [6,50].

Previously, GS and BS patients were classified based on age, the presence of hypercalciuria, polyhydramnios, or growth retardation, and severity. Based on differences in the metabolism of divalent cations, BS and GS can be distinguished by laboratory measurements, which include urinary Ca^2+^ excretion, Ca^2+^/creatinine, and serum Mg^2+^. BS patients tend to have a less severe manifestation compared to GS patients. BS patients have increased urinary calcium excretion and fractional excretion rates, whereas serum magnesium levels are within the normal range [49]. To differentiate between BS and GS, a thiazide test was proposed using a cohort of genetically proven GS and BS patients given oral administration of hydrochlorothiazide (1 mg/kg to 50 mg) [51]. In patients with GS, fractional chloride excretion showed very little change (<2.3%) as the defect was in the thiazide-sensitive Na/Cl cotransporter (NCCT). In contrast, patients with BS showed an increased response to HCO_3_ administration, which led to a more pronounced excretion. Despite the successful differentiation between GS and BS, the test is not routinely recommended due to the increased risk of volume depletion [51].

Due to the rare nature of the syndrome, there is only limited clinical information on treatment. Therapeutically, BS patients urgently need sufficient fluid and electrolyte replacement. For newborns, it is important to control the risk of pre-renal failure due to imbalances in fluid homeostasis. The classic pharmacological therapy for BS patients includes the administration of potassium chloride, prostaglandin inhibitors (indomethacin), and aldosterone antagonists (spironolactone) [52,53].

The symptoms of BS patients are initially treated with the help of potassium supplementation. Also, potassium-sparing diuretics such as spironolactone, eplerenone, or amiloride are often prescribed. According to the literature, these diuretics lead to reduced aldosterone levels, which subsequently increases serum potassium and, hence, counteracts metabolic alkalosis. In cases of persistent hypokalemia and or other side effects of medications, potassium-sparing diuretics, renin–angiotensin system blockers, or NSAIDs are also prescribed. Combinations of these medications are also recommended in certain cases [44,47,54]. Angiotensin-converting enzyme inhibitors (ACE inhibitors) are used to correct low K^+^ levels or to counteract proteinuria [54,55].

Table 1 provides an overview of the distinct gene variants, clinical symptoms, and treatment options for GS/BS patients.

## 4. Aging and RAAS

Aging is a biological process that affects all organizational levels of an organism. The aging process is considered one of the major risk factors for the most prevalent diseases worldwide [57]. The hallmarks of aging contribute to processes leading to the aging phenotype, which include epigenetic alterations, chronic inflammation, dysbiosis, deregulated nutrient sensing, mitochondrial dysfunction, genomic instability, telomere attrition, loss of proteostasis, deregulated macro-autophagy, stem cell exhaustion, altered intercellular communication, and cellular senescence [58,59,60]. Cellular aging is the result of the accumulation of damaged macromolecules, which are chemically altered by ROS. ROS impairs the integrity and function of mitochondria, resulting in, for example, reduced ATP generation [61].

The RAAS includes several systematic processes for the regulation of blood pressure. This is a consequence of increasing peripheral resistance or vascular tone and cardiac contractility, as well as enhanced water and sodium reabsorption, increased aldosterone secretion, and facilitated catecholamine release from sympathetic nerve terminals [1]. Regulation of hypertension and extracellular volume is regulated by angiotensin II (ANG II) as the biological effector of RAAS [62,63,64]. ANG II preferentially binds two subtypes of angiotensin II receptors: AT1 (AGTR1) and AT2 (AGTR2). AGTRs are expressed in various segments of the nephron in the distal tubule, collecting duct, and renal vasculature [65,66,67,68,69,70]. The known physiological and pathological effects are induced via the AT1 receptor. In humans, AGTR1 receptors are expressed in a wide variety of organs. They are present in glomerular mesangial cells, proximal and distal tubular epithelial cells, medullary interstitial cells, and renal vasculature [71,72,73]. The binding of ANG II to AGTR1 activates four classical signaling cascades, including phospholipase A2, phospholipase C, phospholipase D, and L-type calcium channels, and normally blocks the adenylate cycle.

ANG II binding to renal AGTR1 initiates vasoconstriction, sodium reabsorption, protein synthesis, and cellular growth [73,74].

In comparison, the activation of AGTR2 mediates K^+^ channel activity via the protein-coupled receptors and activates protein tyrosine phosphatase (PTP), which leads to a reduction in the activities of mitogen-activated protein kinase (MAPK) and extracellular signal-regulated kinases ERK1 signaling pathways [75,76]. The activation of AGTR2 leads to increased bradykinin production, which induces vasodilation via the nitric oxide (NO)/cyclic guanosine monophosphate (GMP) pathway [73]. ANG II binding to AGTR2 initiates vasodilation, differentiation, apoptosis, anti-proliferation, and sodium transport imbalance of membrane ionic effects [77].

Both types of receptors are involved in the regulation of renal hemodynamics, tubular function, renal cellular growth, and matrix formation [73].

During activation of RAAS, binding of ANG II to AGTR1 leads intracellular nicotinamide adenine dinucleotide phosphate (NADPH) oxidase to generate superoxide anions and promotes uncoupling of endothelial NO synthase, which in turn decreases NO availability, and increases ROS production. The promotion of oxidative stress and DNA damage mediated by ANG II is tightly regulated. However, if uncontrolled, ANG II-mediated ROS production occurs; this leads to cellular senescence-associated mitochondrial dysfunction, hypertrophy, inflammation, and fibrosis in the kidneys, heart, and brain [78,79]. ANG II-mediated signaling via AGTR1 additionally leads to shortening of telomeres and ultimately glomerular mesangial cell senescence but this can be counteracted by losartan [80].

The mammalian ortholog of the silent information regulator (Sir2) of yeast, SIRT1, is a nicotinamide adenine dinucleotide (NAD)+-dependent class III histone deacetylase involved in many aging-associated processes such as apoptosis, cell differentiation, development, stress response, metabolism, and tumorigenesis [41,81,82]. SIRT1 expression is reduced in senescent mesenchymal stem cells, whereas its over-expression slows the onset of senescence and loss of differentiation capacity [83]. SIRT1 is associated with longevity, and in a hypertensive patient, low SIRT1 levels lead to an acceleration of the aging process [84,85]. The same is true for endothelial progenitor cells induced by ANG II, which presumably have reduced SIRT1 expression [86,87]. To counteract this, studies have been conducted investigating the role of polyphenol resveratrol in relation to hypertension [88,89]. Resveratrol (3,5,4′-trihydroxystilbene) is a polyphenol found in red wine that has several beneficial effects on cardiovascular disease [90,91]. In mammalian cells, resveratrol can promote various cellular functions and signaling pathways including longevity, cell cycle regulation, apoptosis, DNA damage repair, and muscle differentiation through the activation of SIRT1 [92,93]. In a study carried out by Miyazaki et al., resveratrol intake was shown to reduce AGTR1 expression in vascular smooth muscle cells [88]. Furthermore, resveratrol has been shown to downregulate AGTR1 expression via SIRT1 and has beneficial effects on hypertension [88]. Currently, several approaches can be employed to counteract premature aging such as caloric restriction, strength training, or nutrition [94,95,96,97,98]. In DNA repair mutant mice, caloric restriction reduced ROS and other reactive compounds, resulting in lower levels of DNA damage [99].

According to a study by the Robert Koch Institute, around every third person in Germany and, therefore, around 20–30 million German citizens are afflicted with high blood pressure [100]. Worldwide, about a fourth of the world’s population is affected. The prevalence of high blood pressure increases with age, with almost two-thirds of people aged 65 and over having a diagnosis of high blood pressure [100]. Globally, hypertension is responsible for ten million deaths each year and is a critical risk factor for cardiovascular disease.

**Figure 3:**
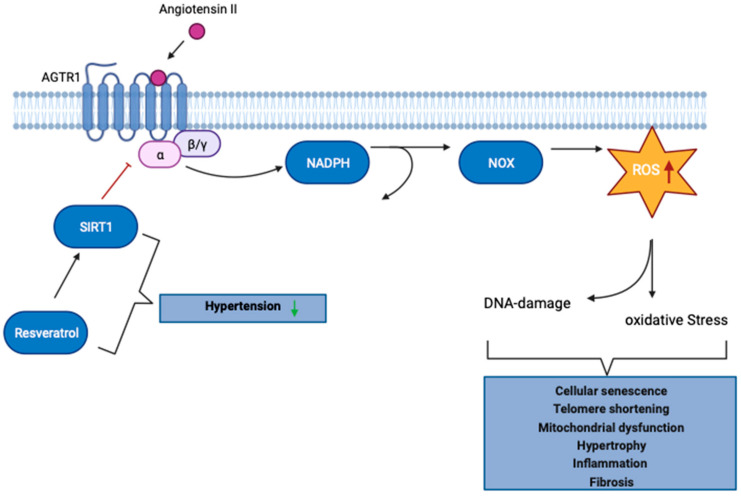
The role of the RAAS in the aging process. Cellular aging and stimulation of RAAS are closely intertwined. Sustained stimulation of angiotensin II (ANG II) receptor type 1 (AGTR1) causes intracellular NADPH oxidase to generate superoxide anions and promote uncoupling of endothelial NO synthase, which in turn decreases NO availability and increases reactive oxygen species (ROS) production. Uncontrolled ANG II-induced ROS production ultimately results in cellular senescence, mitochondrial dysfunction, hypertrophy, inflammation, and fibrosis in the kidney. Sirtuin-1 (SIRT1), involved in many age-associated processes, can counteract the uncontrolled ANG II-induced effects, by regulating the expression of AGTR1. Resveratrol is a natural compound capable of promoting SIRT1 activity and has been shown to have beneficial effects on hypertension. Adapted from “Activation of Protein Kinase C (PKC)”, by BioRender.com (2023). The Figure was generated from https://app.biorender.com/biorender-templates accessed on 7 December 2023.

Multiple key mechanisms including inflammation, oxidative stress, as well as endothelial dysfunction lead to accelerated aging and hypertension. They can occur either independently or together [101,102,103]. Hypertension is an age-associated disease, as illustrated in Figure 3.

### The Hallmarks of Aging in the Etiology of Sodium Deficiency-Associated Diseases

The aging process is one of the main causes of cancer, metabolic disorders, and heart and kidney diseases. Aged tissue is accompanied by a progressive loss of physiological integrity, which leads to dysfunction, and is prone to death. The accumulation of reactive oxygen and nitrogen species (RONS)-induced cellular and organ damage is often associated with aging. RONS are generated by various endogenous and exogenous processes, which are normally neutralized by antioxidant defenses, e.g., lipoic and uric acid and coenzyme Q. When an imbalance occurs between the attack and defense systems, oxidative stress ensues. Oxidative stress is part and parcel associated with various age-related diseases. The formation of RONS can be attributed to endogenous as well as exogenous sources.

**Figure 4:**
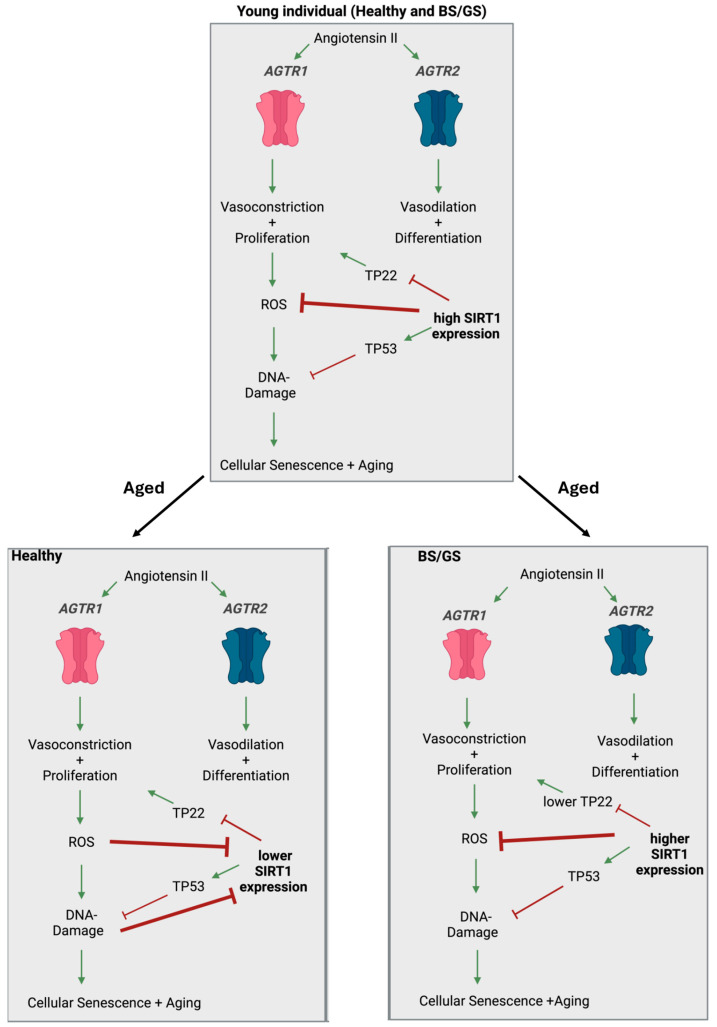
A proposed mechanism of how ROS, SIRT1, and RAAS impinge in Gitelman and Bartter syndromes. Regulation of the renin-angiotensin-aldosterone system (RAAS) is regulated by the octapeptide Angiotensin II (ANGII). In a young individual independent in healthy or in Bartter syndrome (BS)/Gitelman syndrome (GS) patients ANGII binds to either of the two subtypes of Angiotensin II receptors, AT1 (AGTR1) initiates vasoconstriction and proliferation, and AT2 (AGTR2) initiates vasodilation and differentiation. In a healthy but aged individual, such a stimulatory response of AGTR1 leads to increased arteriolar vasoconstriction, proliferation, and reactive oxygen species (ROS) activation which then induces DNA damage, cellular senescence, and acceleration of the aging process. This process can be reversed/minimized by high levels of sirtuin-1 (SIRT1). It is assumed that GS and BS patients have comparable levels of SIRT1 as seen in young individuals. The figure was created with BioRender.com.

Exogenous sources of RONS include tobacco, alcohol, heavy metals, air and water pollution, radiation, etc., which are subsequently converted into free radicals [104]. Endogenous sources include, for example, myeloperoxidase, NADPH oxidase, and ANG II [105]. NADPH is an enzyme complex consisting of several subunits that uses nicotinamide adenine dinucleotide phosphate to produce superoxide anions (O_2_^•−^) [106]. Here, most of the di-oxygen is dismutated into hydrogen peroxide [104]. This can form the highly reactive ROS hydroxyl ion (OH^−^) by a Fenton or Haber–Weiss reaction. In addition, nitric oxide, which is formed from L-arginine by nitric oxide synthase (NOS), is also one of the endogenous sources. This can be divided into three main isoforms: epithelial NOS, neuronal NOS, and inducible NOS. Epithelial NOS is associated with vasodilation and vascular regulation and has a fundamental link with RAAS as well as with tubular disorder diseases such as Gitelman syndrome and Bartter syndrome (Figure 4) [107].

The cortical segments of the nephron have a high density of mitochondria [108,109]. Mitochondria not only play an essential role in the metabolism of the kidney but also signal transmission, and they facilitate stress reactions of kidney cells. Mitochondrial defects such as mutations in nuclear or mtDNA are common causes of mitochondrial dysfunction leading ultimately to the development of multiple kidney diseases [110,111,112]. In detail, permanent mitochondrial dysfunction can lead to inflammation, oxidative stress, loss in cellular functions and structure of renal cells, as well as reduced adenosine triphosphate (ATP) production [108,113,114]. These consequences are also linked to the aging process.

Mutations within mtDNA can result in Gitelman and Bartter syndromes [42,115,116]. Active mitochondria increase ROS levels, thus activating the RAAS. This is associated with increased NCC activity and, thus, with the regulation of DCT-specific NaCl reabsorption [117].

In a healthy person, kidney function often declines, leading to an accumulation of metabolic waste products and excessive electrolytes. Low potassium concentration associated with excessive salt concentration appears to be a highly prevalent cause of hypertension. High sodium intake induces intra-renal and vascular RAAS activation, which ultimately produces pro-inflammatory and pro-fibrotic conclusions [118,119,120].

GS and BS patients have salt wasting and are protected from the well-documented increase in sodium reabsorption and retention promoted by ANG II. 

GS patients have reduced serum phosphate [121], Ca^2+^ reduction [122], and PKC activation, in contrast to a healthy individual [122,123,124]. PKC is a negative regulator of endothelial nitric oxide synthase (eNOS) and has a positive effect on vasodilation in GS and BS patients, as evidenced by increased urinary eNOS expression. Thus, it can be concluded that increased eNOS and NO-mediated vasodilation in GS/BS patients is crucial for the response of endothelial cells to increased flow in the brachial artery [11,125,126].

ANG II induces a reduction in the number of endothelial progenitor cells (EPCs), which are crucial for regulating heart diseases such as hypertension [126]. ANG II binding to AGTR1 leads to induced senescence of EPCs in mice and the development of oxidative stress [12].

In GS/BS patients, senescence, as well as the production of oxidative stress, remains under control at non-deleterious levels. It appears that in GS/BS patients, there is drastically reduced hypertension, cardiac remodeling, and the reduction in oxidative stress and its associated proteins despite constitutive RAAS stimulation secondary to salt wasting [12,125]. Mammalian aging is characterized by somatic mutations and other forms of DNA damage such as chromosomal abnormalities [127]. When these changes occur, the cell cycle is arrested in the G1 phase, which is triggered by TP53, TP22, as well as TP16 [81,128].

Four distinct processes can be initiated depending on the cell type, transient cell cycle arrest associated with DNA repair, apoptosis, senescence, or cell differentiation [129]. A connection between TP53 and Gitelman syndrome is currently not established. According to the literature, p22/PRG1 is a novel target gene of p53 target [130]. Caló et al. reported that GS/BS patients have less oxidative stress and low levels of p22 expression [131]. In relation to p53, this may imply that GS/BS patients have lower p53 activity and, consequently, the activation of p22 is lower [86,127,129].

SIRT1 expression in GS/BS patients tends to be higher than in healthy age-matched individuals; therefore, it can be assumed that there will be a trend towards healthy aging (Figure 4) [10].

Regulation of the renin–angiotensin–aldosterone system (RAAS) is regulated by the octapeptide ANG II. In a healthy young individual or in BS/GS syndrome patients, ANG II binds to either of the two subtypes of angiotensin II receptors, AGTR1 initiates vasoconstriction and proliferation, and AGTR2 initiates vasodilation and differentiation. In a healthy but aged individual, such a stimulatory response of AGTR1 leads to increased arteriolar vasoconstriction, proliferation, and ROS activation, which then induces DNA damage, cellular senescence, and acceleration of the aging process. This process can be reversed/minimized by high levels of SIRT1. It is assumed that GS and BS patients have comparable levels of SIRT1 to those seen in young individuals. The figure was created with BioRender.com.

## 5. Conclusions

Salt intake is a determinant of high blood pressure and as such has an important role in the sensitivity of hypertensive responses. Salt-sensitive individuals show strong hypertension responses compared to BS/GS patients [132,133].

Several diseases are associated with magnesium deficiency, such as Gitelman and Bartter syndromes. The binding of angiotensin II to AGTR1 leads to ROS production and subsequently to cellular senescence associated with mitochondrial dysfunction, hypertrophy, inflammation, and renal fibrosis [78,79,134]. However, none of these hypertensive consequences have been observed to date in GS/BS patients despite their high-salt medication [135,136]. GS and BS patients show downregulation of inflammation-related processes and have reduced oxidative stress and ROS production. Explanations for this may include the reduced release of messenger substances by AGTR1 [137], reduced Ca^2+^ release and PKC activation [137], or higher SIRT1 expression [10]. SIRT1 expression in GS/BS patients tends to be higher than in healthy age-matched individuals; therefore, it can be assumed that there will be a trend towards healthy aging. As these reports are sparse, more studies are needed to confirm these observations.

Another link can be made to immune deficiency in patients with salt-wasting tubulopathies, including GS [138]. Here, the effects of salt wasting may provide a further link between GS/BS and protection against some effects of aging by analysis of the signaling pathways and excreted proteins. The addition of salt as a therapeutic intervention, which is already used as therapy today, could play a crucial role in this process.

These findings should not only apply to GS/BS but could also provide general links to salt-wasting tubulopathies.

## 6. The Future Direction of Research

We previously demonstrated that urine can serve as a non-invasive source of bipotential SIX2-positive renal progenitor cells [139], which can be differentiated into renal tubular cells and podocytes [62,63,139]. These cells can be employed for studying kidney-associated aging processes [81] and ANG II-mediated RAAS activation [62,63], and can be reprogrammed into induced pluripotent stem cells to enable studying the effects of a mutation on other cell types [140].

We are currently isolating SIX2-positive urine-derived progenitor cells from GS and BS patients with the aim of differentiating these into either tubular cells or podocytes, which will be ideal for studying the effect of angiotensin II and the RAAS pathway in these patients.

More recently, iPSCs have been generated from GS patients harboring the common Asian mutations [141,142]. GS and BS patient-derived iPSC will enable far-reaching investigations using revolutionized gene editing methods such as Clustered Regularly Interspaced Short Palindromic Repeats (CRISPR) to correct the mutation(s) in the GS/BS patient-derived iPSC lines to provide isogenic disease and healthy lines, which can be differentiated either to tubular cells or podocytes to study disease mechanisms and also toxicology and drug screening. There are GS and BS knockout mice, which can be used to investigate the link between hypertension, oxidative stress, and aging. With these human cellular and mouse models, the path is now paved for increased efforts in uncovering the underlying etiology leading to Gitelman and Bartter syndromes. These cellular models will enable drug screening/development and toxicology studies, thus leading to better and more targeted treatment options in the near future.

## Figures and Tables

**Table 1:** Comparisons between Gitelman and Bartter syndromes.

Gitelman Syndrom				Bartter Syndrom			
Variant	Gene Mutation/ OMIM	Gene Product	Clinical Symptoms	Variant	Gene Mutation/ OMIM	Gene Product	Clinical Symptoms
**Gitelman syndrome (GS)**	*SLC12A3/* 263800	NCC	• present in later childhood or adulthood with • weakness • hypokalemic alkalosis • hypomagnesemia • hypermagnesemia • hypocalciuria	**Type I** **(neonatal Bartter syndrome/** **hyperprostaglandin E syndrome)**	*SLC12A1/* 601678	NKCC2	• polyhydramnios • prematurity • hypokalemic • hypochloremic alkalosis • nephrocalcinosis with or without concentrating defect
**GS-like syndrome** mtDNA	*MT-TF* /590070 *MT-TI/* 590045	Mitochon-drially encoded TRNA Phen-ylalanine and isoleucine	• dysfunction of oxidative phosphorylation complex IV • reduced maximal mitochondrial respiratory capacity • hypokalemia • hypomagnesemia • arterial hypertension • hypercholesterole-mia	**Type II** (neonatal Bartter syndrome)	*KCNJ1/* 241200	ROMK1	• polyhydramnios • prematurity • transient hyperkalemia and acidosis • hypokalemic hypochloremic alkalosis • nephrocalcinosis, with or without concentrating defect
**EAST syndrome** (SeSAME)	*Kir4.1/* *612780*	KCNJ10	• epilepsy • ataxia, • sensorineural deafness • hypokalemic • hypochloremic alkalosis • muscle cramps • paresthesias • nocturia • salt craving • muscle weakness • fatigue	**Type III** (classic Bartter syndrome)	*CLCNKB/* 607364	CIC-Kb	Variable age at presentation with severity corresponding to type of gene mutation; • hypokalemic • hypochloremic alkalosis
**Other pathogenic variants**		*FXYD2* and *HNF1B*	• sensorineural deafness • epilepsy • ataxia • intellectual disability • diabetes • renal cysts • muscle cramps • paresthesias nocturia • salt craving • muscle weakness • fatigue	**Type IVa** **(Classical Bartter syndrome with sensorineural hearing loss)**	*BSND/* 602522	BARTTIN	• polyhydramnios • prematurity • hypokalemic • hypochloremic alkalosis • sensorineural deafness, with or without concentrating defect
				**Type IVb**	*CLCNKA, CLCNKB/* 613090	CIC-Ka and CIC-Kb	Polyhydramnios, prematurity, hypokalemic hypochloremic alkalosis, sensorineural deafness, with or without concentrating defect
				**Type V** **(autosomal dominant hypoparathyroidism)**	*L125P/* 601199	CASR	Hypocalcemic hypocalciuria, hypokalemic hypochloremic alkalosis, suppressed PTH
**Therapy**	Supplementation of: • potassium • potassium chloride • magnesium			Supplementation of: • potassium chloride • sodium • magnesium			
	High-salt diet						
	Potassium-sparing diuretics: • spironolactone • triamterene			Potassium-sparing diuretics: • spironolactone • amiloride			
	Nonsteroidal anti-inflammatory drug (NAID): • Indometacin			Nonsteroidal anti-inflammatory drug (NAID): • Ibuprofen			
	Renin-angiotensin system blocker: • spironolacton • eplerenon			Renin-angiotensin system blocker: • eplerenone			

Gitelman syndrome (GS) and Bartter syndrome (BS) have distinct genetic mutations. BS is divided into five subtypes depending on the affected gene (I, II, III, IVa, IVb, V). NKCC2: furosemide-sensitive sodium–potassium–2-chloride cotransporter; ROMK: renal outer medullary potassium channel; CLC-Kb: chloride channel Kb; CLC-Ka: chloride channel Ka; CaSR: calcium-sensing receptor; Gitelman syndrome is caused by biallelic inactivating mutations in the SLC12A3 gene. NCC: thiazide-sensitive sodium chloride cotransporter. MT-TF: mitochondria-encoded tRNA-Phe (UUU/C)); KCNJ10: Potassium Inwardly Rectifying Channel Subfamily J Member 10; FXYD2: FXYD Domain Containing Ion Transport Regulator 2; HNF1B: hepatocyte nuclear factor 1-beta. Modified version from Fulchiero et al. [56].
